# Do partial AZFc deletions affect the sperm retrieval rate in non-mosaic Klinefelter patients undergoing microdissection testicular sperm extraction?

**DOI:** 10.1186/s12894-020-00587-5

**Published:** 2020-02-27

**Authors:** I-Shen Huang, Richard J. Fantus, Wei-Jen Chen, James Wren, Wei-Tang Kao, Eric Yi-Hsiu Huang, Nelson E. Bennett, Robert E. Brannigan, William J. Huang

**Affiliations:** 1 Department of Physiology, School of Medicine, National Yang-Ming University, Taipei, Taiwan; 2 Department of Urology, School of Medicine, National Yang-Ming University, Taipei, Taiwan; 3 Department of Urology, Taipei Veterans General Hospital, No 201, Section 2, Shipai Rd, Taipei, 112 Taiwan, Republic of China; 4 Department of Urology, School of Medicine, Shu-Tien Urological Research Center, National Yang-Ming University, Taipei, Taiwan; 5 Section of Urology, Department of Surgery, University of Chicago Medicine, 5841 S. Maryland Avenue, Chicago, IL 60637 USA; 6 Division of Male Reproductive Surgery and Men’s Health, Department of Urology, Northwestern University Feinberg School of Medicine, NMH/Arkes Family Pavilion Suite 2300, 676 N Saint Clair, Chicago, IL 60611 USA; 7 Department of Urology, Shuang Ho Hospital, Taipei Medical University, No.291, Zhongzheng Rd., Zhonghe District, New Taipei City, 23561 Taiwan; 8 Graduate Institute of Clinical Medicine, Taipei Medical University, New Taipei City, Taiwan

**Keywords:** Klinefelter, AZFc deletion, Age, Microdissection testicular sperm extraction

## Abstract

**Background:**

The purpose of this study is to evaluate the prognostic factors for sperm retrieval and determine if Y chromosome deletion is associated with deleterious effects on spermatogenesis in non-mosaic Klinefelter patients. Whether Y chromosome deletion determines the sperm retrieval rate in non-mosaic Klinefelter patients has not yet been addressed.

**Methods:**

We retrospectively collected medical records of azoospermic patients from Sep 2009 to Dec 2018, and enrolled 66 non-mosaic 47, XXY patients who were receiving mTESE. The predictive values of patients age, serum follicle-stimulating hormone (FSH), luteinizing hormone (LH), testosterone, prolactin, estradiol and Y chromosome deletion were assessed for successful sperm recovery.

**Results:**

Testicular sperm recovery was successful in 24 (36.4%) of 66 men. The mean age (36.0 vs. 36.6 years), and levels of FSH (30.0 vs 36.9 IU/L), LH (17.7 vs 21.9 IU/L), testosterone (2.4 vs. 2.1 ng/ml), prolactin (9.1 vs. 8.8 ng/ml), and estradiol (19.4 vs. 22.3 pg/ml) did not show any significant difference when comparing patients with and without successful sperm retrieval. Partial deletion of azoospermic factor c (AZFc) was noted in 5 (20.8%) of 24 patients with successful sperm retrieval, including three *b2/b3* and two *gr/gr* deletion cases, whereas 4 (9.5%) of 42 patients with unsuccessful sperm retrieval were noted to have AZFc partial deletion (one *b2/b3*, one sY1206 and two *gr/gr* deletion), though the difference was not statistically significant (*p* = 0.27).

**Conclusion:**

According to present results, age and AZFc partial deletion status should not be a deterrent for azoospermic males with non-mosaic Klinefelter syndrome to undergo mTESE.

## Background

According to recommendations from the American Society for Reproductive Medicine (ASRM) and European Association of Urology (EAU), cytogenetic analysis is suggested for patients with azoospermia or oligozoospermia. However, the threshold of sperm count recommended for karyotype analysis differs between the two associations: spermatozoa count below 10 million/mL from the EAU and spermatozoa count below 5 million/mL from the ASRM [1, 2]. The main purpose of performing cytogenetic analysis for patients with subfertility or infertility is to provide proper counselling before assisted reproductive technology and inform couples of possible inheritances of paternal genetic disorders in their offspring.

Genetic mutation or chromosome abnormality is more common in infertile males than in the general population, and investigations have shown that the percentage of chromosome abnormalities and Y chromosome microdeletions is about 2–6% and 3–5% [3, 4] for oligospermic patients, 13–15% and 6–16% for non-obstructive azoospermia (NOA) patients [5, 6]. Among all chromosome abnormalities, Klinefelter syndrome (KS) is the most common numerical chromosome abnormality found in infertile men with an incidence rate of about 1-in-500 to 1-in-1000 male birth [7]. The majority of cases with 47, XXY non-mosaic Klinefelter karyotype will present as hypergonadotropic hypogonadism and azoospermia, regardless of age, as a sign of primary testicular failure [8]. However, aside from the non-mosaic form, there is a strong probability of finding sperm in semen with mosaic form (46, XY/47, XXY) of KS, depending on the level of XXY cell line mosaicism in the gonads [9]. In general, about 90% of patients have uniform 47, XXY karyotype (pure KS) in which an extra X chromosome is present in somatic and germ cells, while 10% of KS can present with higher-grade of X chromosome aneuploidies (48 or more chromosomes) as well as mosaicism (47, XXY/46, XY) [7]. Mosaic KS men tend to be more androgenized than their non-mosaic KS counterparts in terms of hormone profile and sperm count in ejaculate, representing better fertility potential, especially in low-grade mosaicism form [9].

The azoospermia factor (AZF) region on the Y chromosome long arm is crucial for spermatogenesis and is known for its unique nearly identical direct and indirect repeated palindromic sequences. In addition to KS, the deletion of AZF is another cause for male infertility [10]. Removal of the spermatogenesis candidate gene located in the AZFa or AZFb, which is responsible for production of male gametes, namely the USP9Y, DDX3Y, RBMY and PRY genes, will inevitably lead to spermatogenic failure and therefore is not recommended for microdissection testicular sperm extraction (mTESE) due to the lack of identifiable regions of spermatogenesis in the testes [11]. The result of semen analysis of men with AZFc deletion varies in comparison with azoospermia found in AZFa and AZFb men, ranging from azoospermia to moderate oligozoospermia. Despite causing detrimental effects in sperm production, AZFc deletion is still regarded as a good prognostic factor for azoospermic men; the sperm retrieval rate (SRR) is up to 70% in this population [12].

The incidence of AZFc deletion is higher than incidence of AZFa or AZFb deletion in men, because the Y chromosome AZFc region is predisposed to intrachromosomal non-allelic homologous recombination owing to its composition of large repeat units called “amplicons” [13].

Rearrangement of the AZFc region, including partial deletions, duplications and deletions followed by duplication(s) has been reported [13]. Deletion of the entire AZFc locus on the Y chromosome by means of removing 3.5 Mb segment, including eight multi-copy gene families, is consensually considered a cause for infertility [10, 14]. As for AZFc partial deletion, Gr/gr deletion is the most common type, followed by b2/b3 and b1/b3 deletions. In particular, whether gr/gr deletion causes deleterious effects on spermatogenesis is still an ongoing debate. According to previous literature, the frequency of gr/gr deletion is only higher in certain azoospermia or oligozoospermia populations in Italy and China when compared to the control [15, 16].

Researchers have attempted to find predictors for successful sperm retrieval in KS men; some have demonstrated that testosterone levels after medical treatment, including aromatase inhibitors, clomiphene or human chorionic gonadotropin [17] and age at time of mTESE are reliable prognostic factors [18]. Therefore, they have recommended that patients receive mTESE for sperm retrieval under the age of 35 [18]. To date, due to the limited number of KS men, there are a paucity of articles discussing sperm retrieval rate by mTESE. Moreover, previous study populations did not include Han-Chinese or whether Y chromosome microdeletion may serve as a prognostic factor for possible sperm retrieval. Some clinicians may infer the outcome of sperm retrieval is affected by Y chromosome microdeletion in Klinefelter patients. Our study aimed to analyze predictive factors for sperm retrieval in Han-Chinese KS men and discuss the association for Y chromosome microdeletion with successful sperm retrieval.

## Methods

### Studied populations

We retrospectively reviewed the period between September 2009 to Dec 2018. A total of 66 non-mosaic Klinefelter patients who were receiving microdissection testicular sperm extraction were enrolled in our study. All subjects were genetically ethnic Han-Chinese from Taiwan. Each patient underwent a detailed examination to identify the etiology of azoospermia, including a detailed history, physical examination, two consecutive semen analyses, hormone profile (follicle stimulating hormone (FSH), leutinizing hormone (LH), testosterone and prolactin), chromosome karyotyping, and Y chromosome microdeletion. DNA samples of 107 fertile controls who had fathered at least one child were obtained from 2 medical centers in Taiwan as described by Lin et al. in 2007 [14]. This study was performed according to the Taipei Veterans General Hospital approved Institutional Review Board protocol (IRB number: 2018–03-002CCF).

### DNA extraction

Genomic DNA was extracted from peripheral blood using the QIAamp DNA minikit (Qiagen, Hilden, Germany) according to the manufacturer recommendations. In brief, 20 μl Qiagen protease was pipetted into the bottom of a 1.5 ml microcentrifuge tube, then up to 200 μl whole blood or buffy coat was added to the microcentrifuge tube. After the addition of 200 μl buffer AL to the sample, the sample was thoroughly mixed by vortex mixing and was incubated at 56 °C for 10 min until cell lysis was complete. Two hundred microliters of ethanol (100%) were added to precipitate the DNA. The mixture was carefully applied to the QIAamp spin column, placed inside a 2 ml collection tube, and centrifuged at 8000 rpm, 6000×g for 1 min. The QIAamp Spin column was then placed in a new collection tube, and 500 μl of buffer, AW1 was added and centrifuged. This step was repeated with the buffer AW2 and a high spin was given for 3 min to dry the column membrane. The spin column was subsequently placed in a clean 1.5 ml microcentrifuge tube, and the DNA filtrate was recovered with 200 μl of buffer AE. Following centrifugation, DNA was eluted and stored at − 20 °C until used.

### Cytogenetic analysis

Chromosome analysis was performed using conventional trypsin-Giemsa banding technique. Twenty metaphases were analyzed for each patient, and in cases of suspected mosaicism, the number of metaphases was increased to a total of 50 for analysis. To achieve a more detailed analysis, a 500–550 band level was used for evaluation.

### Yq classical deletions and AZFc subdeletions

**Fig. 1:**
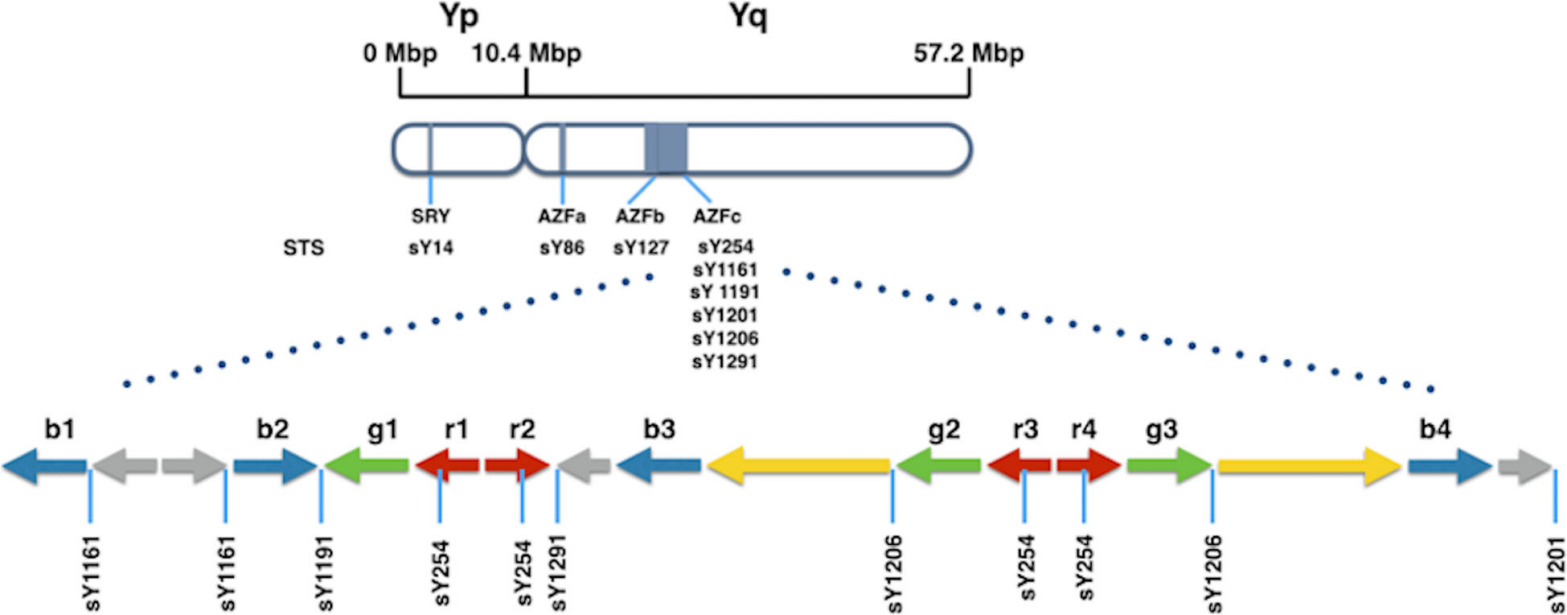
STS markers for Y chromosome microdeletion

Y microdeletion screening in Klinefelter patients was performed by multiplex polymerase chain reaction (PCR) that detects markers in the AZF regions, the sex determining region Y (SRY) gene, and the Y homologous gene pair zinc-finger Y (ZFY) [19]. The sequence-tagged sites (STS) selected are listed in Fig. 1 and Table 1. Six AZFc deletions (sY254, sY1161, sY1191, sY1201, sY1206, and sY1291) were used to identify the type of partial AZFc deletions in all subjects. In addition, DNA from fertile males and female served as positive and negative controls, respectively. Ambiguities in the multiplex PCR assays were resolved by following up with single PCR assays.

**Table 1:** Sequence of 10 sets of primer employed to identify Y-chromosome microdeletions

STS/gene	Primer sequence	Product size (bp)	Region
SRY-F	5′ - GAA TAT TCC CGC TCT CCG GA - 3′	495	NRY
SRY-R	5′ - GCT GGT GCT CCA TTC TTG AG - 3’		
ZFY-F	5′ - ACC RCT GTA CTG ACT GTG ATT ACA C - 3’	472	NRY
ZFY-R	5′ - GCA CYT CTT TGG TAT CYG AGA AAG T - 3’		
sY86-F	5′ - GTG ACA CAC AGA CTA TGC TTC - 3’	320	AZFa
sY86-R	5′ - ACA CAC AGA GGG ACA ACC CT - 3’		
sY127-F	5′ - GGC TCA CAA ACG AAA AGA AA - 3’	274	AZFb
sY127-R	5′ - CTG CAG GCA GTA ATA AGG GA - 3’		
sY254-F	5′ - GGG TGT TAC CAG AAG GCA AA - 3’	400	AZFc
sY254-R	5′ - GAA CCG TAT CTA CCA AAG CAG C - 3’		
sY1161-F	5′ - CGA CAC TTT TGG GAA GTT TCA - 3’	377	AZFc
sY1161-R	5′ - TTG TGT CCA GTG GTG GCT TA- 3’		
sY1191-F	5′ – CCA GAC GTT CTA CCC TTT CG - 3’	385	AZFc
sY1191-R	5′ – GAG CCG AGA TCC AGT TAC CA - 3’		
sY1201-F	5′ – CCG ACT TCC ACA ATG GCT - 3’	677	AZFc
sY1201-R	5′ – GGG AGA AAA GTT CTG CAA CG - 3’		
sY1206-F	5′ – ATT GAT CTC CTT GGT TCC CC - 3’	394	AZFc
sY1206-R	5′ – GAC ATG TGT GGC CAA TTT GA - 3		
sY1291-F	5′ – TAA AAG GCA GAA CTG CCA GG - 3’	527	AZFc
sY1291-R	5′ – GGG AGA AAA GTT CTG CAA CG - 3’		

### Microdissection testicular sperm extraction

A small incision (3 cm) was made longitudinally on the median raphe, and carried down to the fascia. The testis were mobilized after opening the tunica vaginalis as described in 1999 [20]. We performed a longitudinal incision on the testis instead of open it widely in an equatorial plane along the mid portion of the testis. An operating microscope with 20x~24x magnification allow the surgeon to identify larger and opaquer seminiferous tubules, which are most likely to contain sperm. Tissue with possibility of spermatogenesis was removed and sent for real time analysis by experienced embryologists in our reproductive lab for further intracytoplasmic sperm injection (ICSI).

### Statistical analysis

Outcome variables (age, serum FSH, LH, testosterone, prolactin and estradiol) of azoospermic non-mosaic KS men with successful surgical sperm retrieval were compared to those with unsuccessful sperm retrieval using the Student’s t test. The distributions of AZFc partial deletion of Klinefelter patient with or without sperm retrieval and the frequency of AZFc partial deletion comparing azoospermic non-mosaic Klinefelter men with controls were analyzed by the Fisher’s exact test. In these tests, values of *p* < 0.05 were regarded as statistically significant.

## Results

### Azoopsermic non-mosaic Klinefelter men and Y chromosome deletion

The cohort of 66 azoospermic non-mosaic Klinefelter men with chromosome 47, XXY had a mean age of 36.4 ± 5.2 years (mean ± SD). Of these 66 men, 9 men (13.6%) had a deletion of one STS marker. More precisely, we identified 5 men with an absence of sY1291 (possible *gr/gr* deletion), 3 men with an absence of sY1191 (possible *b2/b3* deletion) and 1 with an absence of sY1206 only. Of the 24 patients with successful sperm retrieval, 5 (20.8%) had partial AZFc deletion, including 2 possible *gr/gr* deletion and 3 possible *b2/b3* deletion cases (Table 2). Partial AZFc deletion is identified in 4 (9.5%) of 42 patients without sperm retrieval by mTESE, (one possible *b2/b3*, one sY1206, and two possible *gr/gr* deletion), though the difference of Y chromosome deletion was not statistically significant between the successful and non-successful sperm retrieval groups (*p* = 0.27).

**Table 2:** Demographic profile of nonmosaic Klinefelter men with positive and negative mTESE outcome

	Overall Mean ± SD	mTESE (+) Mean ± SD	mTESE (−) Mean ± SD	*p* value
Number	*n* = 66	*n* = 24	*n* = 42	
Age (yr)	36.4 ± 5.2	36.0 ± 4.9	36.6 ± 5.4	0.67
FSH (IU/L)	34.3 ± 17.0	30.0 ± 13.1	36.9 ± 18.6	0.12
LH (IU/L)	20.4 ± 11.7	17.7 ± 9.7	21.9 ± 12.6	0.16
Testosterone (ng/nl)	2.2 ± 1.8	2.4 ± 2.0	2.1 ± 1.6	0.47
Prolactin	9.2 ± 3.7	8.7 ± 3.5	9.5 ± 3.8	0.39
Estradiol (pg/ml)	21.0 ± 9.9	19.5 ± 7.4	21.8 ± 11.0	0.41
Y partial deletion	9 (13.6%)	5 (20.8%)	4 (9.5%)	0.27
gr/gr deletion	4 (6.1%)	2 (8.3%)	2 (4.8%)	0.62
b2/b3 deletion	4 (6.1%)	3 (12.5%)	1 (2.4%)	0.13
sY1206 deletion	1 (1.5%)	0 (0%)	1 (2.4%)	1.00

### Impact of various parameters on mTESE success

Of the non-mosaic KS patients, mTESE was successful in 24 out of 66 patients, representing 36.4% of cases. Table 2 lists the clinical factors analyzed to predict sperm retrieval, stratified by outcome. Age, partial AZFc deletions, and hormone parameter (LH, FSH, testosterone, prolactin, estradiol) were not predictive factors for successful sperm retrieval.

### Effect of age on sperm retrieval

**Fig. 2:**
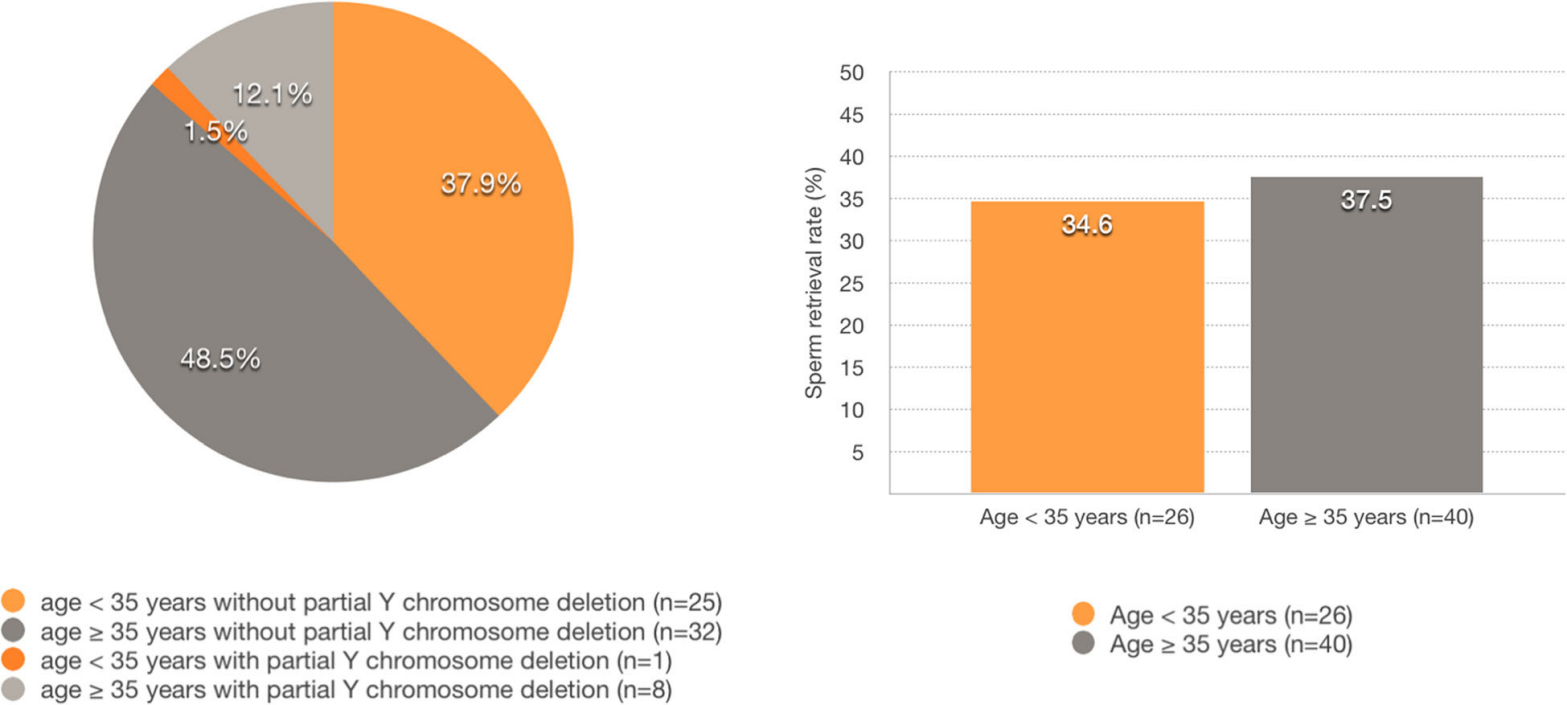
Percentage of KS men below and above 35 with Y chromosome deletion and sperm retrieval rate by mTESE

SRR for mTESE was 34.6% (9/26) at ages below 35 years and 37.5% (15/40) at 35 years and older. Four of 13 KS men older than 40 years of age had successful sperm retrieval; the oldest patient with successful mTESE was 47 years-old (Fig. 2).

### Partial AZFc deletions distribution between the experimental and control groups

The frequency of AZFc partial detection was 13.6% (9/66) in KS men and 6.5% (7/107) in the control, although a higher percentage was seen in KS men, however the difference was not statistically significant (*p* > 0.05). The deletion pattern in 9 KS men with AZFc partial deletion included 4 men with possible *gr/gr* deletion, 4 with possible *b2/b3* deletion, and one with sY1206 deletion. Seven men in the control group with AZFc partial deletion included 3 men with possible *gr/gr* deletion and 4 men with possible *b2/b3* deletion. There was no significant difference between the frequencies of specific AZFc partial deletion pattern by means of possible *gr/gr*, possible *b2/b3*, and sY1206 deletion, respectively (Table 3). Y chromosome partial deletion was detected in 5 KS men with successful sperm retrieval.

**Table 3:** AZFc partial deletion in KS men and controls

	Fertile control	Klinefelter men	*p* value
Total subjects	107	66	
AZFc partial deletion	7	9	0.18
gr/gr deletion	3	4	0.43
b2/b3 deletion	4	4	0.48
sY1206 deletion	0	1	0.38

## Discussion

The extra X chromosome in KS men causes germ cell degeneration and adverse effects to somatic cells, including Leydig and Sertoli cells, resulting in infertility. As a matter of fact, germ cell degeneration take place early in the fetal period, whereas the density and number of seminiferous tubules and mesenchymal tissues remain normal, without obvious pattern change [21]. The hormone levels are unaffected until the onset of puberty, which is when the germ cell depletion, Sertoli cell degeneration, Leydig cell hyperplasia and seminiferous tubule hyalinization initiate and accelerate [22]. Accelerated degeneration of the Sertoli cell is also evidenced by the sign of aberrant nuclear expression of the α-subunit in the Sertoli cells in older teenage KS boys [23]. Based on the concept of early fertility deterioration, sperm banking at a younger age is recommended at puberty, according to their Tanner stage. The results, however, were not promising with regards to KS adolescent semen quality. Sperm in the ejaculate can only be detected in limited cases [8].

If sperm cannot be traced in the semen, using mTESE for testicular sperm retrieval associated with assisted reproductive technology such as ICSI is a recommended option with a satisfactory outcome, and offers these patients the opportunity to father biological children. As shown in several studies, age is a predictive factor for successful sperm retrieval in KS men receiving mTESE; In adult men younger than 35 years old, sperm retrieval rate (SRR) is reported to range from 30 to 77%, with a higher SRR than subjects > 35 years. Consequently, experts recommend performing mTESE under the critical age of 35 or even 30 years to yield a better outcome [18, 24]. However, age as a predictive factor for sperm retrieval was not unanimously proved in other literatures [25, 26]. In our study, age was not a predictive factor for sperm retrieval, and SRR was comparable for KS men under or above the age of 35 (33.3% vs 37.5%, respectively). The mean age of our study population is 36.4 years; only two KS men (age 25 and 27 years) under the age of 30 was evaluated, which will limit the ability to evaluate the influence of age on successful sperm retrieval.

Our Klinefelter cases with older age may be explained by the fact that a majority of cases coming to our center are either referral or consultation for infertility other than delayed puberty, which also implies that most of the Taiwanese KS men remained undiagnosed before marriage. According to Taiwanese government statistics, the average age of marriage for men has increased in the last decade and was 34.2 years in 2015.

*gr/gr* deletion, the most common form of partial AZFc deletion, has been demonstrated a correlation with male infertility in many meta-analysis studies [27, 28]. However, debates rage over whether *gr/gr* deletion and copy number reduction of AZFc genes influence spermatogenesis. The diverse phenotypes in *gr/gr* deletion prevent researchers from drawing definite conclusions on this topic, which is best shown in the Japanese population with 33.9% *gr/gr* deletion men in the control group, and 23.9% in the infertility group, although the controls have unknown fertility status [29]. Based on ethnicity and region, Caucasians (OR = 3.721), people from Europe (OR = 2.465), and people from South Asia (OR = 2.523) with *gr/gr* deletion had a higher risk of male infertility in comparison to those without deletions [28]. But published literatures within the same ethnicity have drawn opposite conclusions. In China, Wu et al. pointed out that *b2/b3* deletion was significantly associated with idiopathic male infertility [30], while in contrast, Yang et al. recorded *gr/gr* deletion, instead of *b2/b3* deletion, serve as a more important anomaly in determining the susceptibility to spermatogenic failure [31].

As a matter of fact, the results may vary without contradiction. Two study populations that were 1500 km apart belonged to different Y chromosome haplogroup, representing distinct paternal lineage. As demonstrated previously, Y lineages as defined by different haplotypes could influence the phenotypical expression of identical *gr/gr* deletion genotype [32]. *Gr/gr* deletions and *b2/b3* have been demonstrated fixed in haplogroup Q1 and haplogroup N; the prevalence of partial deletion will likely mask actual influence from other haplogroups [14, 27, 33, 34]. In the Taiwanese population, Lin et al. found that 14% of men have deletions and duplications of AZFc and that the frequency of deletion vary between different Y haplogroups, ranging from 2.9% in O3e to 100% in N and Q [14]. AZFc partial duplication other than partial deletion increases the risk of spermatogenic failure. Although, in our study, we did not perform quantitative PCR analysis to evaluate DAZ (Deleted in AZoospermia) dosage and duplication in our patients, with regard of the presence of AZFc, partial deletion did not seem to affect SRR.

In Danish and Chinese populations, specific Y haplogroup has been suggested to be an “at risk Y haplogroup” by their over-presentation in the spermatogenic impairment population [31, 35]. Until now, no study has looked into the relationship between different Y haplogroup and SRR in azoospermic cases. Hence, it would be interesting to understand the possible influence of Y haplogroup not only on semen parameters but also on spermatogenesis in the testes.

We examined Y chromosome microdeletions in KS men using 10 sets of primers and detected 9 cases with partial AZFc deletion, including 4 men with possible *gr/gr* deletion, 4 with possible *b2/b3* deletion, and one with sY1206 deletion KS. Our study did not detect complete AZFa, AZFb or AZFc deletion in KS men, which is compatible with the Lin et al. study, suggesting a low percentage of complete deletion pattern in Han Chinese in Taiwan [14]. The incidence of AZFc partial deletion did not differ between the KS men and the fertility control men; the result is consistent with a recent study investigating AZF microdeletion in KS men [36]. However, in a study of Han Chinese KS men in China, the proportion of partial AZFc deletion is higher than our population – 27 (24.3%) of 111 KS men and 11 (11.7%) of the 94 controls compared to our population of 13.6% in KS men and 6.5% in the control [36]. The outcome of our study indicates that the possible *gr/gr* and *b2/b3* deletions are not correlated with KS men in the Taiwan Han-Chinese population, suggesting that although formation of sperm with an extra copy of X chromosome by non-disjunction during meiosis I contributed to paternal error, it did not increase the chance of de novo Y chromosome partial microdeletion.

Previous studies on the preoperative hCG or aromatase inhibitor on KS men have demonstrated varying results, therefore our KS cases were not routinely given hCG or aromatase inhibitors before mTESE [17, 24, 26].. Prior experience with mTESE in KS men with non-obstructive azoospermia reported SRR range from 16 to 70% and cumulative SRR per TESE cycle of 44% [37]. The SRR for KS men above 35 years of age in 3 series were 25, 30 and 50%; it was 37.5% in our study [17, 18, 24].

To our knowledge this is the first article looking into Y chromosome deletion in Klinefelter patients for SRR evaluation. Our results demonstrate that AZFc partial deletion, age, and endocrine variables do not predict sperm retrieval in KS men. Sperm retrieval rate is comparable for men below and above 35 years, and we should not discourage AZFc partial deletion men or men over the age of 35 from accepting mTESE for possible sperm retrieval.

## Conclusion

According to present results, age and AZFc partial deletion status should not be a deterrent for azoospermic males with non-mosaic Klinefelter syndrome to undergo mTESE.

## Data Availability

The data that support the findings of this study are available on request from the corresponding author W.H. The data are not publicly available due to them containing information that could compromise research participant privacy.

## Abbreviations

ASRM: American Society for Reproductive Medicine
AZF: Azoospermia factor
DAZ: Deleted in AZoospermia
EAU: European Association of Urology
FSH: Follicle stimulating hormone
ICSI: Intracytoplasmic sperm injection
KS: Klinefelter syndrome
LH: Leutinizing hormone
mTESE: Microsurgical testicular sperm extraction
NAHR: Non-allelic homologous recombination
NOA: Non-obstructive azoospermia
PCR: Polymerase chain reaction
SRR: Sperm retrieval rate
SRY: Sex determining region Y
STS: Sequence-tagged sites
ZFY: Zinc-finger Y
